# Reply to Letter to the Editor: “Can single CT slices revolutionize personal identification in emergency settings?”

**DOI:** 10.1007/s00330-024-11131-6

**Published:** 2024-10-28

**Authors:** Andreas Heinrich

**Affiliations:** Department of Radiology, Jena University Hospital–Friedrich Schiller University, 07747 Jena, Germany

Thank you very much for the positive feedback on my research [1] and for the constructive comments. I would like to address the points raised:

The limitation mentioned relates specifically to this publication [1], which only examined axial slices. Although axial slices may capture only part of the region due to varying head positions, the large size of the maxillary sinuses usually provides enough matching points. However, the method is also applicable to other slice orientations and 2D/3D reconstructions. A forthcoming publication will demonstrate how maximum intensity projection (MIP) images can also be effectively used for personal identification with this method.

Minimizing metal artifacts is an important area for future research, as these artifacts can reduce or obscure edge contrast, leading to fewer matching points. While maxillary sinuses and ethmoidal cells are rarely affected, metal artifacts can hinder the comparison of orthopantomogram (OPG)-like images from CT data using curved multiplanar reconstruction (MPR) with a large computer vision (CV) database of OPGs. Comparing OPG-like images with a CV database of over 82,000 OPGs demonstrates significant potential for unique identification, which will be further explored in a forthcoming publication.

The call for prospective studies is valid, but certain prerequisites must be met. This retrospective study aimed to showcase the method’s potential. Prospective studies require large CV databases to avoid skewed identification rates, as the sought individual may not be present in the CV database. Therefore, a primary goal is to develop an international CV database with numerous entries [2]. CV features do not allow image reconstruction, ensuring strong data privacy. Patient IDs in the CV database are pseudonymized, with decryption only possible through the image source (the originating clinic), thus maintaining data control with the image creator. Under these conditions, prospective studies will be feasible. Additionally, establishing a threshold score to indicate the likelihood of identification would reduce the need for full database comparisons and be beneficial when the sought identity might not yet be in the CV database. I have already identified the potential of such a threshold in comparisons with OPGs [3, 4] and CT slices.

In conclusion, the method presented shows great promise and potential for practical application. The positive feedback on my work supports the aim to develop an international CV database, facilitate large-scale prospective multicenter studies, and transition from fundamental research to practical implementation. I am sincerely grateful for the feedback, as support letters are essential for securing the funding needed to achieve these ambitious goals.
